# Arthroscopic partial meniscectomy for a degenerative meniscus tear: a 5 year follow-up of the placebo-surgery controlled FIDELITY (Finnish Degenerative Meniscus Lesion Study) trial

**DOI:** 10.1136/bjsports-2020-102813

**Published:** 2020-08-27

**Authors:** Raine Sihvonen, Mika Paavola, Antti Malmivaara, Ari Itälä, Antti Joukainen, Juha Kalske, Heikki Nurmi, Jaanika Kumm, Niko Sillanpää, Tommi Kiekara, Aleksandra Turkiewicz, Pirjo Toivonen, Martin Englund, Simo Taimela, Teppo L N Järvinen, for the FIDELITY (Finnish Degenerative Meniscus Lesion Study) Investigators, Anna Ikonen

**Affiliations:** 1 Department of Orthopaedics and Traumatology, Pihlajalinna Oyj, Tampere, Pirkanmaa, Finland; 2 Finnish Centre for Evidence-Based Orthopedics (FICEBO), University of Helsinki, Helsinki, Finland; 3 Department of Orthopedics and Traumatology, Töölö Hospital, Helsinki University Hospital, Helsinki, Uusimaa, Finland; 4 Department of Orthopedics and Traumatology, University of Helsinki, Helsinki, Finland; 5 Centre for Health and Social Economics – CHESS, National Institute for Health and Welfare, Helsinki, Finland; 6 Pohjola Hospital, Turku, Finland; 7 Department of Orthopaedics and Traumatology, Kuopio University Hospital, Kuopio, Finland; 8 Department of Orthopedics and Traumatology, Helsinki University Hospital, Helsinki, Finland; 9 Department of Orthopedics and Traumatology, Central Finland Central Hospital, Jyväskylä, Finland; 10 Department of Medicine, Tartu Ulikool, Tartu, Tartumaa, Estonia; 11 Medical Imaging Center, Tampere University Hospital, Tampere, Finland; 12 Department of Orthopedics, Lund University, Lund, Sweden; 13 Clinical Epidemiology Unit, Orthopaedics, Lund University, Lund, Sweden

**Keywords:** osteoarthritis, meniscus

## Abstract

**Objectives:**

To assess the long-term effects of arthroscopic partial meniscectomy (APM) on the development of radiographic knee osteoarthritis, and on knee symptoms and function, at 5 years follow-up.

**Design:**

Multicentre, randomised, participant- and outcome assessor-blinded, placebo-surgery controlled trial.

**Setting:**

Orthopaedic departments in five public hospitals in Finland.

**Participants:**

146 adults, mean age 52 years (range 35–65 years), with knee symptoms consistent with degenerative medial meniscus tear verified by MRI scan and arthroscopically, and no clinical signs of knee osteoarthritis were randomised.

**Interventions:**

APM or placebo surgery (diagnostic knee arthroscopy).

**Main outcome measures:**

We used two indices of radiographic knee osteoarthritis (increase in Kellgren and Lawrence grade ≥1, and increase in Osteoarthritis Research Society International (OARSI) atlas radiographic joint space narrowing and osteophyte sum score, respectively), and three validated patient-relevant measures of knee symptoms and function (Western Ontario Meniscal Evaluation Tool (WOMET), Lysholm, and knee pain after exercise using a numerical rating scale).

**Results:**

There was a consistent, slightly greater risk for progression of radiographic knee osteoarthritis in the APM group as compared with the placebo surgery group (adjusted absolute risk difference in increase in Kellgren-Lawrence grade ≥1 of 13%, 95% CI −2% to 28%; adjusted absolute mean difference in OARSI sum score 0.7, 95% CI 0.1 to 1.3). There were no relevant between-group differences in the three patient-reported outcomes: adjusted absolute mean differences (APM vs placebo surgery), −1.7 (95% CI −7.7 to 4.3) in WOMET, −2.1 (95% CI −6.8 to 2.6) in Lysholm knee score, and −0.04 (95% CI −0.81 to 0.72) in knee pain after exercise, respectively. The corresponding adjusted absolute risk difference in the presence of mechanical symptoms was 18% (95% CI 5% to 31%); there were more symptoms reported in the APM group. All other secondary outcomes comparisons were similar.

**Conclusions:**

APM was associated with a slightly increased risk of developing radiographic knee osteoarthritis and no concomitant benefit in patient-relevant outcomes, at 5 years after surgery.

**Trial registration:**

ClinicalTrials.gov (NCT01052233 and NCT00549172).

## Introduction

Arthroscopic partial meniscectomy (APM) is one of the most common orthopaedic surgical procedures; over half a million surgeries are performed annually in the USA, and 1.1 million APM surgeries were performed in the UK between 1997 and 2017.^1 2^ Most procedures are performed in middle-aged and older patients.^1^ Proponents of APM point to improved knee pain and function and quality of life after surgery as evidence of its efficacy. However, 13 trials, rigorously summarised in 2017 in a clinical practice guideline^3^ based on two systematic reviews,^4 5^ provided strong evidence that APM offers, at best, only little short- to medium-term benefit for most patients with degenerative knee disease compared with sham surgery or non-surgical management.

Some clinicians argue that an untreated meniscus tear increases the risk of knee osteoarthritis (OA). However, evolving consensus—based on observational data—suggests that APM is associated with increased risk of progression of knee OA and subsequent need for “corrective” surgery (high tibial osteotomy or total knee replacement).^6 7^ The respective roles of the underlying degenerative process and the potential harmful effect of arthroscopic surgery are challenging to disentangle in the above noted study designs due to confounding by indication.^8^

The risk of serious complications within 90 days of APM is likely low (<0.3%).^9^ However, any indication of possible *long-term* harmful effect should raise concern, given APM is unlikely to confer substantial benefit. Thus, to study whether APM (resection of torn meniscus tear) per se accelerated or delayed development of knee osteoarthritis in patients with an arthroscopically-verified degenerative tear of the medial meniscus, we carried out a pre-registered 5 year follow-up of our placebo-surgery controlled FIDELITY trial (Finnish Degenerative Meniscus Lesion Study).^10^ The second objective of this analysis was to assess the long-term efficacy of APM on knee symptoms and function.

## Patients and methods

We conducted a multicentre, randomised, participant- and outcome assessor-blinded, placebo-surgery controlled efficacy trial involving participants aged 35–65 years with knee symptoms over 3 months, consistent with degenerative medial meniscus tear and unresponsive to conventional conservative treatment, and no advanced knee osteoarthritis. Patients were recruited from five orthopaedic centres in Finland during the period from December 2007 through January 2012. All patients had a suspicion of a medial meniscus tear based on symptoms and clinical tests, a tear that was later verified on both MRI and knee arthroscopy. Patients with an obvious traumatic onset of symptoms or a recent history of a locked knee were excluded. On entering the study, participants were informed that they would be allowed to consider a reoperation ≥6 months after the procedure if they did not have adequate relief of symptoms.

All participants had a diagnostic knee arthroscopy and were then (during the same operation) assigned to either APM or placebo surgery. For the randomization, we used sequentially numbered, opaque, sealed envelopes prepared by a statistician with no involvement in the clinical care of participants in the trial. Randomization was performed in a 1:1 ratio with a block size of 4. Study site, age (35–50 years or 51–65 years), sex, and the absence or presence of minor degenerative changes on a radiograph (Kellgren-Lawrence grade 0 or 1, respectively) were used to stratify allocation.

The participants, all caregivers, and those assessing the outcomes were blinded to the treatment assignment. Participants completed questionnaires at 2, 6, 12, 24, 36, 48, and 60 months postoperatively. At the 24 and 60 month follow-up, participants had a standardised clinical examination performed by an independent orthopaedic surgeon who was unaware of treatment allocation.

Our primary research questions—on the development of knee osteoarthritis (NCT01052233) and on the efficacy of APM on knee symptoms and function (NCT00549172)—were registered separately in the ClinicalTrials.gov database.

We have previously published the trial protocol^10^ and 12 and 24 month follow-up findings.^11–13^ The trial protocol was approved by the institutional review board of the Pirkanmaa Hospital District (R06157). The trial was conducted in accordance with the Declaration of Helsinki. All participants gave written informed consent.

### Interventions

Arthroscopic evaluation included recording the presence of intra-articular pathology (meniscus tears, loose bodies and characterisation of lesions to the tibiofemoral and patellofemoral chondral surfaces) according to the International Cartilage Repair Society (ICRS) cartilage injury classification scale^14^ and the International Society of Arthroscopy, Knee Surgery and Orthopaedic Sports Medicine (ISAKOS) classification of meniscal tears.^15^

During the APM, the damaged and loose parts of the meniscus were removed with the use of arthroscopic instruments until solid meniscal tissue was reached, with preservation of as much of the meniscus as possible. No other surgical procedure was performed. For the placebo surgery, simulated APM mimicked the sensations and sounds of APM. Participants were kept in the operating room for the same amount of time required to perform APM.

In both the APM and the placebo surgery groups, postoperative care was delivered according to a standard protocol. All participants received the same walking aids and instructions for the same graduated home-based exercise programme.

### Radiographic outcomes

Initially, we registered an increase of one grade or more in the Kellgren and Lawrence knee osteoarthritis grading (dichotomous outcome: Yes or No) as our only primary outcome for the assessment of radiographic knee osteoarthritis (NCT01052233). The Kellgren and Lawrence scale is a semi-quantitative instrument (ordered categorical grades 0–4) to assess the severity of radiographic tibiofemoral knee osteoarthritis.^16^ Patients who had undergone an osteotomy or a total knee replacement during follow-up were considered to have progressed radiographically (dichotomous outcome: Yes). We recruited additional expertise on the assessment of knee osteoarthritis to our research group (ME and AT) before any longitudinal radiographic readings and data analysis were made, and we decided to include another primary outcome: radiographic progression based on the sum of marginal tibiofemoral osteophyte grades and tibiofemoral joint space narrowing grades (according to the atlas developed by the Osteoarthritis Research Society International (OARSI); continuous outcome, hypothetical range 0–18). We documented our additional decisions in the trial statistical analysis plan.^17^ The OARSI atlas is a semi-quantitative instrument (ordered categorical grades 0–3) that assesses the severity of joint space narrowing and osteophytes, respectively, in knee osteoarthritis.^18^

All paired (baseline and 5 year) knee radiographs were initially read longitudinally by one experienced musculoskeletal radiologist (JK) who was blinded to treatment allocation and clinical data, and unblinded to the sequence of the radiographs. The reviewers of our statistical analysis plan^17^ recommended carrying out the analyses using two readers. We therefore recruited two new experienced musculoskeletal radiologists (NS and TK) to complete a new set of readings independently. The new readers were blinded to treatment allocation and clinical data, and unblinded to the time sequence of the radiographs. The readers initially met in person to calibrate their interpretation of grades on a test dataset before scoring the trial images independently. Any disagreements were resolved by consensus for all images (outcomes).^17^

### Patient-relevant outcomes

To assess the efficacy of APM on knee symptoms and function (NCT00549172), our primary outcomes were the Western Ontario Meniscal Evaluation Tool (WOMET), the Lysholm knee score, and knee pain after exercise, all at 60 months after surgery. The WOMET^19^ is a meniscus-specific health-related quality-of-life instrument (HRQoL), validated specifically for patients with a degenerative meniscal tear.^20^ The Lysholm knee score is a validated, condition-specific outcome measure.^21 22^ WOMET and Lysholm scores each range from 0 to 100, with 0 indicating the most severe symptoms and 100 indicating the absence of symptoms. Knee pain after exercise (during the preceding week) was assessed on an 11-point numerical rating scale (NRS) ranging from 0 (no pain) to 10 (extreme pain).

As secondary outcomes, we (1) assessed the frequency of patients in the two treatment groups who did not have adequate relief of symptoms and whose treatment-group allocation was therefore unblinded (‘unblindings’), (2) queried the presence of mechanical symptoms using the locking domain question of the Lysholm knee score,^21^ (3) assessed satisfaction and improvement, (4) registered serious adverse events, and (5) assessed development of knee osteoarthritis according to the American College of Rheumatology (ACR) Clinical Criteria (dichotomous outcome: Yes or No).^23^ To assess mechanical symptoms, we asked patients to choose which of the following best reflected the status of their knee: (i) no locking or catching, (ii) catching sensations but no locking, (iii) occasional locking, (iv) frequent locking, or (v) locked at present. For satisfaction, participants responded to the following questions: “Are you satisfied with your knee at present?” and “Is your knee better than before the intervention?” on a 5-point Likert scale. We classified “Very satisfied” or “Satisfied” as satisfied, and “Neither satisfied nor dissatisfied”, “Dissatisfied” and “Very dissatisfied” as dissatisfied.^24^ For improvement, we considered the responses “Much better” and “Better” to indicate improvement, and “Unchanged”, “Worse” or “Much worse” to indicate no improvement.

### Blinded data interpretation

We interpreted the results of the trial according to a blinded data interpretation scheme.^25^ As two members of the Writing Committee (RS and PT) had previous access to the data, they recused themselves from making any interpretations. A statistician provided the Writing Committee with blinded results from the analyses, with the two arms labelled A and B. The Writing Committee then considered the interpretation of the results until a consensus was reached and agreed in writing on all alternative interpretations of the findings. We recorded the minutes of this meeting (online supplementary appendix 1), which was signed by all members of the Writing Committee. After agreement was reached, the trial statistician revealed the randomisation code and the interpretation corresponding to the correct treatment allocation was chosen. The draft of the manuscript was then finalised.

10.1136/bjsports-2020-102813.supp1 Supplementary data

### Patient involvement

There was no active patient involvement in the design of the study or in the recruitment to, or conduct of, the study. One of the main outcome measures (the WOMET) was initially developed with a patient-centred approach. The items included in the final version of the questionnaire were those identified by patients to have the most significant impact on their quality of life.^19^ When the results of this 5 year follow-up are published, a lay information flyer with final results will be sent to the recruiting centres for dissemination to the trial participants.

### Statistical methods

The trial was originally designed to ascertain whether APM was superior to placebo surgery for treating patients with knee pain and a degenerative meniscus tear. The study was designed to detect a minimal clinically important improvement in the WOMET and Lysholm scores (defined as improvements of at least 15.5 and 11.5 points, respectively) and in the score for knee pain after exercise (improvement of at least 2.0 points), as described previously.^10^

Baseline characteristics were analysed with the use of descriptive statistics. We used logistic regression to analyse the primary and secondary binary outcomes. The model was adjusted for the baseline randomisation stratification factors (age (35–50 years or 51–65 years), sex, and absence or presence of minor degenerative changes on a radiograph (Kellgren and Lawrence grade 0 or 1)). To obtain adjusted risk differences from the logistic model, the method of standardisation was used.^26^ We did not adjust for study site in the logistic regression analysis due to the low number of participants in some centres and the anticipated sparse data. A sensitivity analysis including the study site as a covariate was performed.

We used linear regression to analysis the OARSI sum score, adjusted for randomisation stratification factors and baseline value of the sum score. For the primary analysis of the patient-relevant outcomes assessed at baseline, 2, 6, 12, 24, 36, 48, and 60 months we used a mixed linear regression model. Patient was included as random effect, and time point (2, 6, 12, 24, 36, 48, and 60 months), treatment arm (APM or placebo), time × treatment interaction, and randomisation stratification factors (age (35–50 years or 51–65 years), sex, absence or presence of minor degenerative changes on a radiograph (Kellgren and Lawrence grade 0 or 1), and study centre) were included as fixed effects. The model was adjusted for baseline values of the respective outcome variable.

All statistical analyses were performed on an intention to treat basis. As the frequency of crossover was low (n=8), we did not perform a per-protocol analysis. For the structural outcomes, where five persons could *not* be analysed due to missing data, the analysis was performed on full analysis set^27^ (ie, according to intention to treat principle), excluding participants with missing data. All inferential results are reported with 95% confidence interval (95% CI). Stata 15 (StataCorp 2017, Stata Statistical Software: Release 15, College Station, TX: StataCorp LLC) was used for all statistical analyses.

## Results

**Figure 1:**
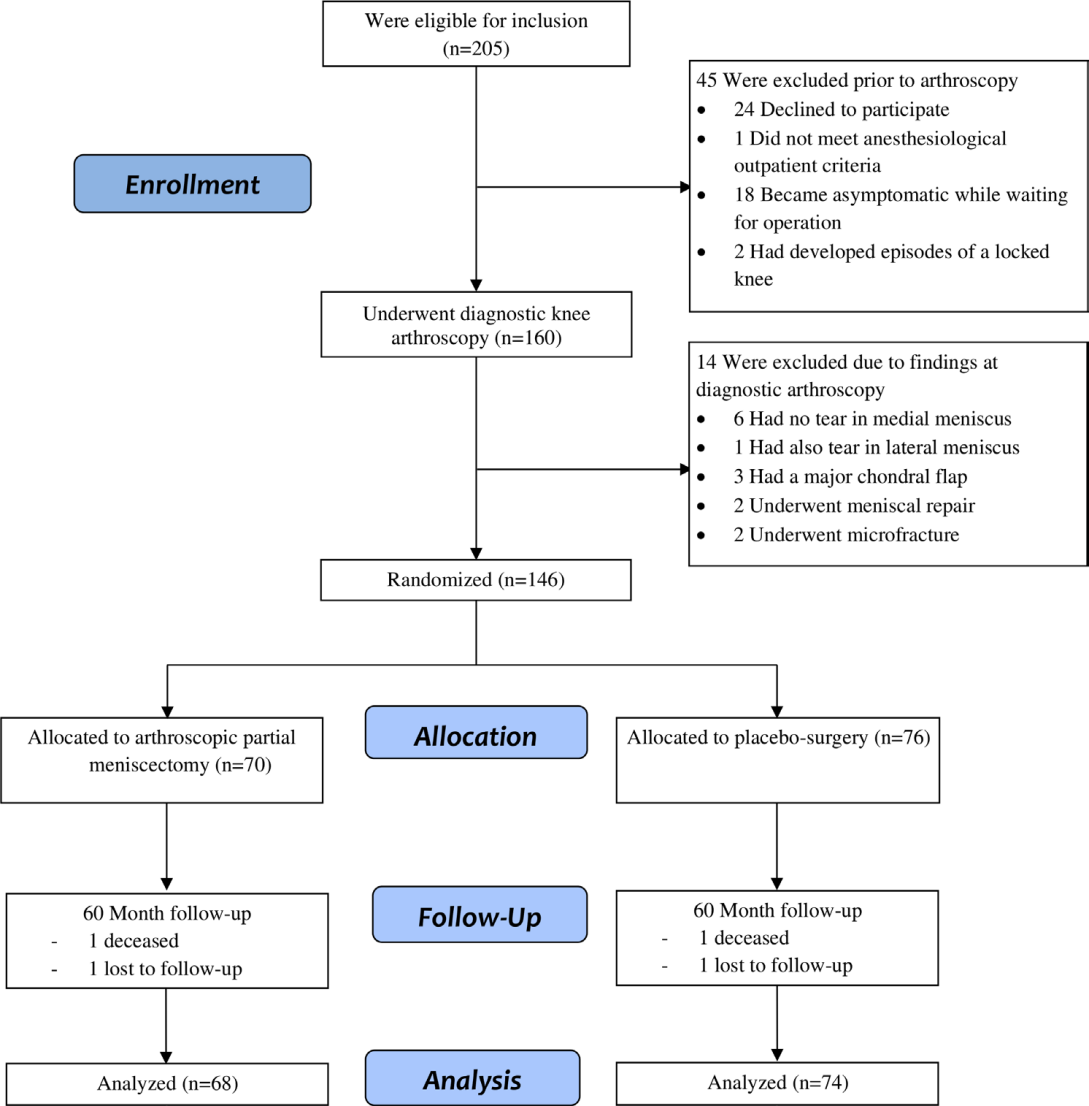
Study flowchart.

Of the 205 eligible patients, 146 were randomised; 70 were assigned to APM and 76 to placebo surgery (figure 1). The baseline characteristics of the two groups were similar (table 1). At the 60 month follow-up, four participants (2.7%) were lost to follow-up (two not responding to contact attempts and two deceased), and one participant completed the questionnaires but did not attend a clinical visit. Of the four lost to follow-up, two participants were from the APM group and two participants were from the placebo surgery group.

**Table 1:** Baseline characteristics of the participants allocated to APM or placebo surgery

	APM (n=70)	Placebo surgery (n=76)
Sex		
Female	28 (40)	29 (38)
Male	42 (60)	47 (62)
Age (years)	52.1±6.9	52.0±7.2
Body mass index (kg/m^2^)	26.9±4.0	27.9±4.0
Duration of symptoms (months)	10 (3–50)	10 (3–47)
Kellgren-Lawrence grade*		
0	18 (26)	25 (33)
1	40 (57)	37 (49)
2	12 (17)	14 (18)
OARSI sum score†	1.9±1.2	1.7±1.3
Meniscal tests		
Positive McMurray test‡	16 (23)	15 (20)
Pain provoked by forced flexion and compression	50 (71)	59 (78)
Pain provoked by palpation at the joint line	63 (90)	74 (97)
Symptoms of catching or locking	32 (46)	37 (49)
WOMET score§	56.4±17.3	52.8±18.1
Lysholm score¶	60.2±14.7	60.1±14.6
Pain after exercise (VAS)**	5.8±2.0	6.1±2.0

Values are numbers (percentages), means±SD or medians (ranges).

*Kellgren and Lawrence scale is a radiographic classification of the severity of knee osteoarthritis. Grade 0 denotes no osteoarthritis, grade 1 possible osteoarthritis, and grade 2 mild osteoarthritis. Scoring based on a consensus reading of two experienced musculoskeletal radiologists blinded to treatment allocation and clinical data.

†The sum of marginal tibiofemoral osteophyte grades and tibiofemoral joint space narrowing (JSN) grades based on the atlas by the Osteoarthritis Research Society International (OARSI) (continuous outcome, hypothetical range 0–18).

‡Results of a McMurray test are positive if a “click” over the medial tibiofemoral joint line is felt by the examiner during flexion and extension of the knee under varus stress.

§The Western Ontario Meniscal Evaluation Tool (WOMET) contains 16 items addressing three domains: 9 items addressing physical symptoms; 4 items addressing disabilities with regard to sports, recreation, work, and lifestyle; and 3 items addressing emotions. The score indicates the percentage of a normal score; therefore, 100 is the best possible score, and 0 is the worst possible score.

¶The Lysholm knee score is based on an eight-item questionnaire designed to evaluate knee function and symptoms in activities of daily living. Scores range from 0 to 100; higher scores indicate less severe symptoms.

**Knee pain after exercise (during the preceding week) was assessed on a rating scale of 0 to 10, with 0 denoting no pain and 10 denoting extreme pain.

APM, arthroscopic partial meniscectomy; VAS, Visual Analogue Scale.

**Figure 2:**
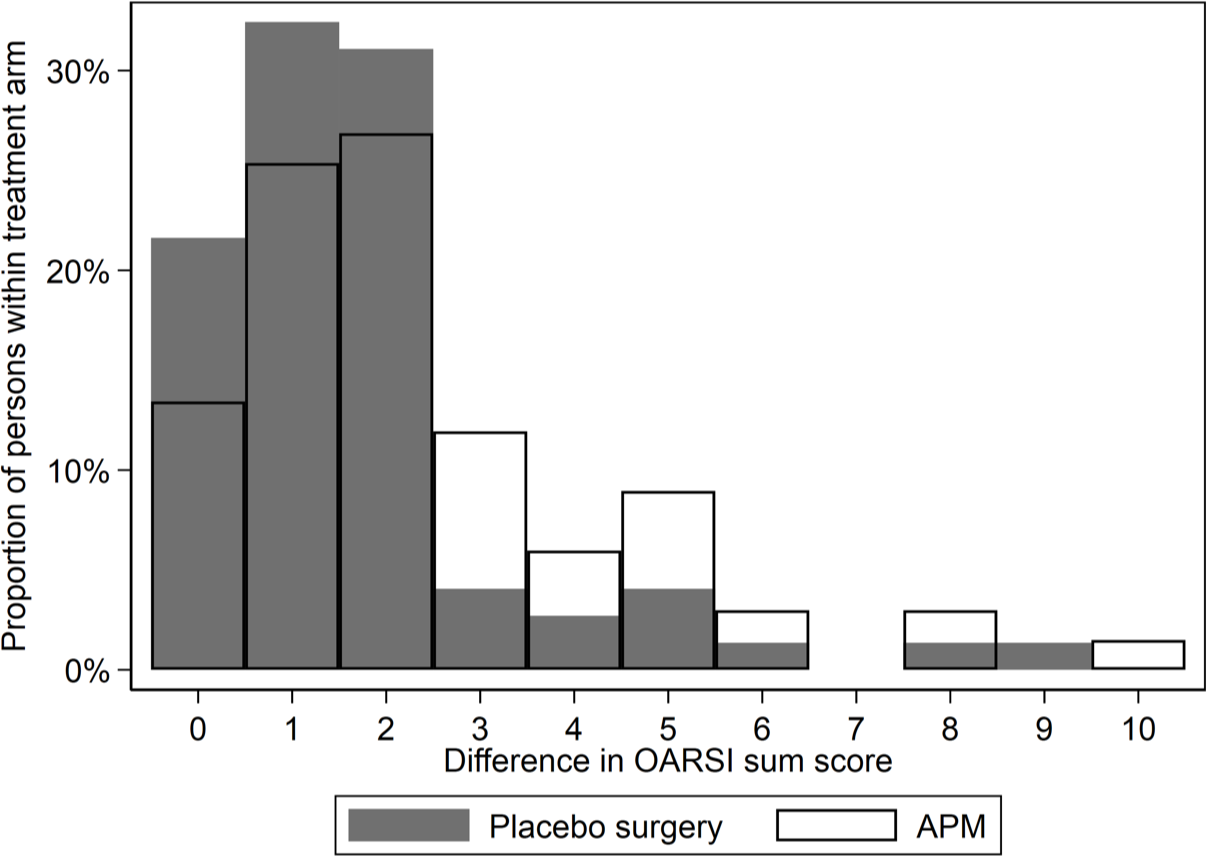
Change in OARSI sum score from baseline to 5 year follow-up in the APM (blank bars) and placebo surgery (dark bars) groups. The x-axis shows the difference between 5 year and baseline OARSI sum score, while the y-axis shows the percentage of participants with each change score, per treatment arm. The higher the bars are at the right end of the x-axis, the more participants with more advanced progression of OA (higher OARSI score). APM, arthroscopic partial meniscectomy; OA, osteoarthritis; OARSI, Osteoarthritis Research Society International.

At 5 years after surgery, 72% (48 of 67) in the APM group and 60% (44 of 74) in the placebo surgery group had at least one grade progression in radiographic tibiofemoral knee OA. The adjusted absolute risk difference was 13% (95% CI −2% to 28%). The adjusted absolute difference in the OARSI sum score was 0.7 (95 CI % 0.1 to 1.3), with more progression in the APM group (figure 2). These findings remained essentially unchanged in a sensitivity analysis that included study site as a covariate.

**Figure 3:**
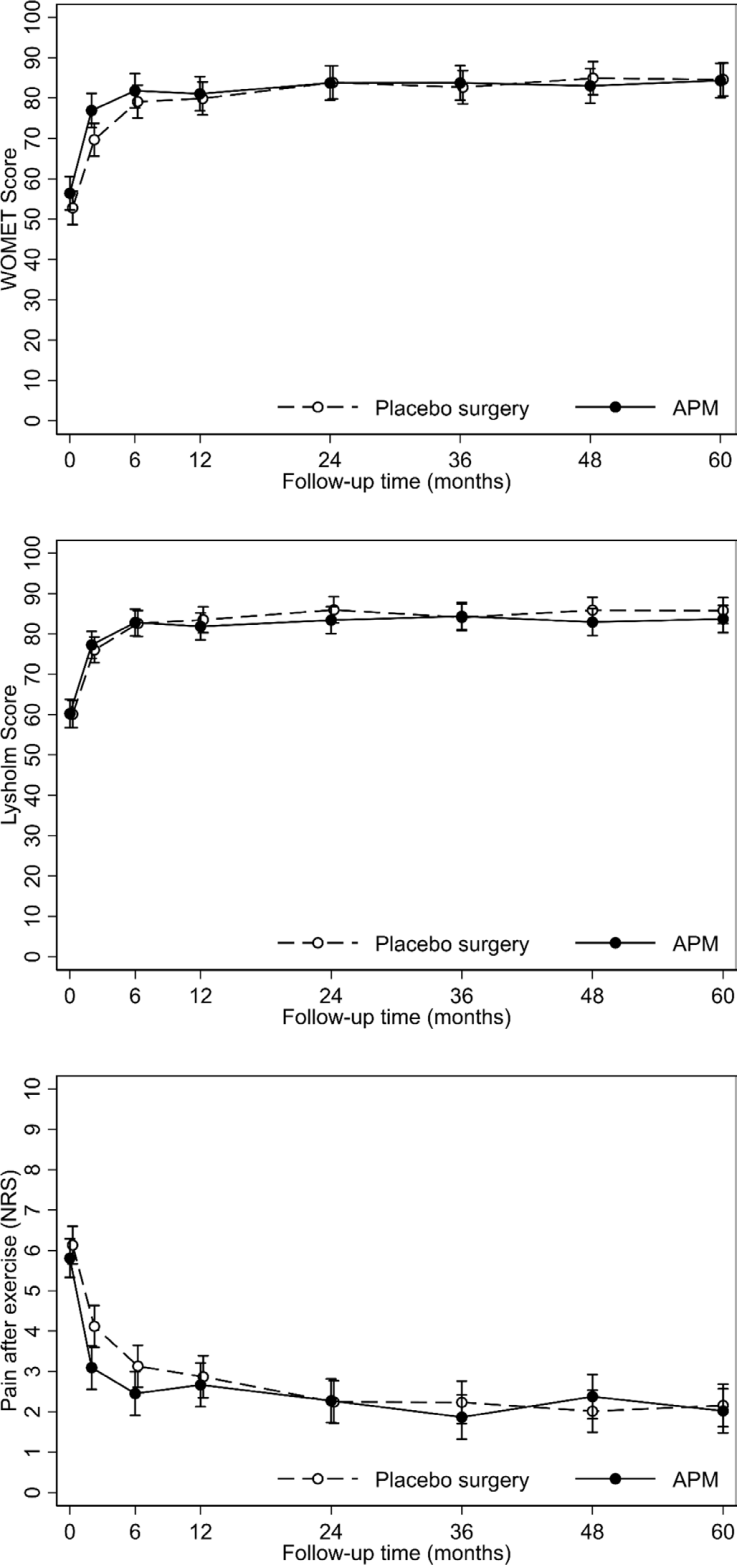
Mean values with 95% confidence intervals in all three primary scores during the 60 month follow-up for both groups. APM, arthroscopic partial meniscectomy.

Both groups reported sustained improvement in knee symptoms and function. There were no relevant between-group differences: adjusted absolute mean differences (APM vs placebo surgery), −1.7 (95% CI −7.7 to 4.3) in WOMET, −2.1 (95% CI −6.8 to 2.6) in Lysholm knee score, and −0.04 (95% CI −0.81 to 0.72) in knee pain after exercise, respectively (table 2 and figure 3).

**Table 2:** Primary patient-relevant outcomes of the trial at 60 month follow-up.

Primary outcomes (adjusted)	APM (n=68)	Placebo surgery (n=74)	Between-group difference in improvement from baseline
WOMET score*	84.3 (80.1 to 88.6)	84.6 (80.5 to 88.7)	−1.7 (−7.7 to 4.3)
Lysholm knee score†	83.7 (80.3 to 87.1)	85.8 (82.6 to 89.0)	−2.1 (−6.8 to 2.6)
Knee pain after exercise‡	2.0 (1.5 to 2.6)	2.2 (1.6 to 2.7)	−0.04 (−0.81 to 0.72)

Values are presented as means (95% CI).

*The Western Ontario Meniscal Evaluation Tool (WOMET) contains 16 items addressing three domains: 9 items addressing physical symptoms; 4 items addressing disabilities with regard to sports, recreation, work, and lifestyle; and 3 items addressing emotions. The score indicates the percentage of a normal score; therefore, 100 is the best possible score, and 0 is the worst possible score.

†The Lysholm knee score is based on an eight-item questionnaire designed to evaluate knee function and symptoms in activities of daily living. Scores range from 0 to 100; higher scores indicate less severe symptoms.

‡Knee pain after exercise (during the preceding week) was assessed on a rating scale of 0 to 10, with 0 denoting no pain and 10 denoting extreme pain.

APM, arthroscopic partial meniscectomy.

Eight participants (12%) in the APM group and eight (11%) in the placebo surgery group reported symptoms severe enough to result in unblinding of group allocation. In both groups, most participants were satisfied (78% in APM vs 84% in placebo surgery) and reported improvement (81% vs 88%, respectively) or return to normal activities (table 3). A higher proportion of the APM group reported mechanical symptoms (adjusted absolute risk difference: 18%, 95% CI 5% to 31%).

**Table 3:** Secondary outcomes of the trial at 60 month follow-up.

Outcome	APM (n=68)	Placebo surgery (n=74)	Risk difference with 95% CI*
Treatment group unblinding	8 (12)	8 (11)	0.01 (−0.09 to 0.12)
Reoperations	7 (10)	8 (11)	0.00 (−0.11 to 0.10)
Arthroscopy	4 (6)	7 (9)	Not applicable
HTO/TKR	3 (4)	1 (1)	Not applicable
Satisfied patients	53 (78)	61 (84)	−0.06 (−0.19 to 0.08)
Improved patients	55 (81)	64 (88)	−0.07 (−0.19 to 0.05)
Returned to normal activities	53 (78)	54 (76)	−0.02 (−0.16 to 0.12)
Serious adverse events†	0	0	
Mechanical symptoms	20 (29)	9 (12)	0.18 (0.05 to 0.31)
Clinical OA according to ACR criteria	5 (8)	6 (9)	−0.01 (−0.09 to 0.08)

Missing data: Treatment-group unblindings: 0; Reoperations: 0; Satisfied: 5; Improved: 5; Returned to normal activities: 7; Mechanical symptoms: 4; Clinical OA: 9.

Descriptive values are numbers (percentage).

*Estimates derived from an adjusted logistic regression model using the method of standardisation to derive risk differences.

†There were no serious adverse events attributable to index surgeries between 24 and 60 months of follow-up. The only serious adverse event encountered was a knee infection after dental procedure in the APM group at 4 months after surgery.

ACR, American Colleague of Rheumatology; APM, arthroscopic partial meniscectomy; HTO, high tibial osteotomy; OA, osteoarthritis; TKR, total knee replacement.

No serious adverse events related to the trial interventions were observed during follow-up from 24 to 60 months.

## Discussion

APM was associated with a slightly increased risk of progression of radiographic knee osteoarthritis without any additional benefit on knee pain, other symptoms or function compared with placebo surgery.

### Comparison with other studies

Concern about the possible detrimental ‘downstream’ effects of APM on knee cartilage was originally prompted by observational data suggesting that both a meniscal tear and prior APM were independent risk factors for radiographic osteoarthritis.^28 29^ A registry-based observational cohort of patients who underwent APM lent further support to the contention that APM per se may increase the risk of OA.^7^ Most recently, two separate analyses of the MeTeOR (Meniscal Tear in Osteoarthritis Research) randomised trial comparing APM with exercise therapy for patients with knee osteoarthritis and a meniscal tear have been published. The first, reporting the progression of MRI-based osteoarthritis markers over the first 18 months, suggested that patients undergoing APM had greater advancement of osteoarthritis than those treated non-operatively.^30^ The second, a 5 year follow-up reporting both patient-reported outcomes and the incidence of total knee replacements (TKR), found no between-group difference in knee pain or function, but a greater likelihood of TKR was observed (HR 2.0, 95% CI 0.8 to 4.9) for participants randomised to APM, compared with those randomised to physical therapy.^31^ Of note, the as-treated analysis of these data suggested that those exposed to APM over the follow-up period had a fivefold increased risk of TKR as compared with those allocated to physical therapy (HR 4.9, 95% CI 1.1 to 20.9). Given that these analyses were adjusted for baseline grade of osteoarthritis, it seems likely that the findings are not due to greater pre-APM radiographic severity. Another recently published 5 year follow-up of a randomised trial reported radiographic deterioration in 60% of patients in the surgery group and 37% in the non-surgery group (p=0.060).^32^ However, all this evidence is subject to considerable uncertainties, as the observational data are prone to confounding by indication while the trial data^30 32^ are hampered by high rates of crossover (around 25–30%) from non-operative treatment to surgery during the follow-up and high loss to follow-up (around 30%).

Confounding by indication is likely to occur in observational studies when a particular intervention is linked to certain selection criteria that may be linked with the outcome of interest. For example, in an observational study looking into the effects of APM on progression of osteoarthritis, patients who have more severe symptoms, which may hypothetically be linked to faster structural progression, may be more likely to be selected for APM. Therefore, the observational study design might falsely lead investigators to conclude that APM is causing knee osteoarthritis.

### Strengths of this study

The elementary difference between our FIDELITY trial and the other trials that have assessed the benefits of APM on patients with knee pain attributed to a torn meniscus is the randomised, placebo-surgery controlled efficacy trial design. A placebo comparison group is generally considered essential in surgical trials with *subjective endpoints*,^33^ as it enables one to distinguish between the treatment effect specifically attributable to the critical therapeutic element of surgery (here, resection of the torn part of the meniscus) and the profound non-specific (and placebo) effects related to the act of surgery in itself.^34–37^ A placebo comparator is also similarly advantageous in teasing out the respective roles of the underlying degenerative process and any potentially harmful effect of the surgical procedure.

Previous *unblinded* trials comparing APM to various non-operative therapies^38–40^ have reported high crossover rates from initial non-operative treatment to surgery. Crossovers have generally been attributed to persisting symptoms and thus interpreted as evidence in support of the superiority of APM over non-operative treatments. However, this interpretation is prone to biases. Patients and researchers may have disproportionate expectations of the benefits of different treatments. When patients are told at entry about the possibility of surgical treatment but then allocated to non-operative treatment, so called ‘failed opportunity’ may mean patients with residual symptoms are less satisfied with their care and subsequently driven to seek surgery.^41^ The bias may also work in the opposite direction. The authors of the MeTeOR trial speculated that the fivefold increase in the risk of TKR for participants ultimately treated with APM may due to the fact that participants who had APM were more familiar and comfortable with the process of undergoing surgery and may have been more inclined to select TKR when symptoms persisted.^31^ Knowledge about prior treatment may also have a subconscious influence on the outcome assessors or the surgeons assessing the severity of residual symptoms.

### Limitations of this study

Regarding the concerns related to crossover in our trial, the frequency of unblindings due to persisting symptoms was similar in the APM and placebo surgery groups (11% and 12%, respectively). The proportions of participants who accurately guessed whether they had undergone a placebo procedure was similar in the two groups.^11^ Crossovers still pose a risk of bias in this trial. Participants who crossed over from placebo surgery to APM were exposed to the potentially negative effects of APM, but remained in their primary allocation group for the statistical analyses (according to the intention to treat principle). Therefore, crossovers may have biased the observed difference towards the null, and our risk estimates for developing knee osteoarthritis due to APM may be underestimated.

Radiographic assessment of knee osteoarthritis is inherently prone to uncertainty due to the subjective nature of the method.^42^ The reviewers of our statistical analysis plan^17^ also raised concerns, and we asked two experienced musculoskeletal radiologists to read all radiographs. In our trial, consensus versus single reader yielded the same results, reassuring us that the main finding of our radiographic analyses—that APM led to a slightly increased risk of developing radiographic knee osteoarthritis—is robust (Online supplemental appendix 2). The acquisition protocol of the knee radiographs dictated that standardised and weightbearing, semi-flexed, bilateral digital radiographs were obtained.^43^ However, not all centres used a mechanical positioning device or fluoroscopy to fix the flexion angle. The flexion angle was not uniform across the whole sample and all time points, which means that minor changes in joint space width must be interpreted cautiously. The Kellgren and Lawrence scale and OARSI atlas are osteophyte-driven grading systems and, as such, are more resilient to variability in the assessment of the joint space width. Possible confounding related to radiograph measures should be equally distributed between the treatment groups, and accordingly our estimates on the magnitude of the between group effects should be valid.

10.1136/bjsports-2020-102813.supp2 Supplementary data

### Conventional wisdom versus FIDELITY findings

Concerns^44–46^ have been expressed regarding the alleged limited generalisability of the FIDELITY trial.^11–13^ It is true that we excluded patients with a true ‘traumatic’ onset of symptoms, which is at odds with the conventional wisdom that ‘traumatic’ tears would be most suitable for surgical resection. However, patients with traumatic meniscal tears may not experience greater improvements in patient-reported outcomes after APM than patients with degenerative tears.^47^

Our trial has been criticised as being “selected” or not representative of “usual APM patients”,^48^ and we agree. We carefully crafted our eligibility criteria to ensure we included patients who were *most likely to benefit* from APM. The efficacy design ensures that our estimates are generalisable: failure to find APM efficacious under these ‘ideal’ circumstances makes it less likely that effectiveness could be proven in routine settings.^49–51^

We have been criticised for recruiting patients with symptoms not attributable to a meniscal tear.^46^ In patients with a degenerative knee disease, knee symptoms are not necessarily attributable to meniscus tissue. Symptoms may originate from other processes or tissues such as bone marrow lesions.^52^ Our eligibility screening reflected the contemporary clinical approach to diagnosing meniscus tear: careful history, standardised clinical examination including all conventional meniscus provocation tests, standard imaging (x-rays and MRI), and arthroscopy to verify the tear.

The presence of mechanical symptoms (sensation of knee catching or locking) is commonly considered a valid indication for arthroscopic surgery.^46 53–55^ This assertion is premised on the rationale that these symptoms are due to a joint structure lodging between the gliding articular surfaces. However, our data (table 3) and a number of other studies show that middle-aged and older patients reporting the presence of mechanical symptoms represent particularly poor candidates for APM.^56–58^ Arthroscopic surgery is indicated for patients with a true locked knee (inability to extend their knee fully) caused by certain types of meniscus tears (eg, bucket-handle tear). However, patients with a true locked knee represent a small subset of the cohort of middle-aged and older patients currently undergoing arthroscopic meniscal knee surgery.^59^

## Conclusions and policy implications

APM was associated with potential harm in the form of a slightly increased risk of development of radiographic knee osteoarthritis and no concomitant benefit in knee pain, other symptoms or function.

**What are the findings?**

- Arthroscopic partial meniscectomy (APM) provided no more benefit for knee symptoms or function than placebo surgery. Arthroscopic partial meniscectomy was associated with a slightly increased risk of developing radiographic knee osteoarthritis at 5 years after surgery.

**How might it impact on clinical practice in the future?**

- Despite robust evidence that APM offers no clinically-relevant benefits for middle aged and older patients with persistent knee pain, the procedure remains one of the most common orthopaedic surgeries.
- Our findings corroborate a body of evidence from observational studies and unblinded randomised controlled trials suggesting an increased risk of developing knee osteoarthritis after APM. We strongly encourage clinicians and patients to consider alternatives to APM for managing knee symptoms.

10.1136/bjsports-2020-102813.supp3 Supplementary data

10.1136/bjsports-2020-102813.supp4 Supplementary data
