# Supplementary material for: Arthroscopic partial meniscectomy for a degenerative meniscus tear: a 5 year follow-up of the placebo-surgery controlled FIDELITY (Finnish Degenerative Meniscus Lesion Study) trial
Source: Br J Sports Med. 2020 Aug 27;54(22):1332–9. doi: 10.1136/bjsports-2020-102813 (PMC7606577; doi:10.1136/bjsports-2020-102813)
Supplement: Supplementary data [file bjsports-2020-102813supp001.pdf]

FIDELITY Finnish Degenerative Meniscus Lesion Study

FIDELITY Trial: Minutes of the Blinded Data Interpretation meeting of the 5-yr follow-up

Interpretation of Blinded Data, Statement of Interpretation

Clinical Epidemiology Unit, Orthopaedics, Department of Clinical Sciences Lund, Lund University, Lund, Sweden

Date: June 26, 2019 at 10:30.

Present:
Aleksandra Turkiewicz, chair
Martin Englund
Pirjo Toivonen
Simo Taimela
Raine Sihvonen
Velocity Hughes
Teppo Järvinen, secretary

Background assumptions (theoretical basis/commitments and previous knowledge for data analysis and scientific goal of being objective and free of preconceptions)

1) This superiority RCT has two research objectives:
    a. to assess the long-term efficacy of arthroscopic partial meniscectomy (APM) (vs. placebo-surgery) in adult (age 35 to 65 years) patients with a degenerative meniscus tear; and
    b. to determine the effects of APM on the development/progression of radiographic and clinical knee osteoarthritis (OA) in patients with a degenerative meniscus tear.

   Both research objectives were registered in the ClinicalTrials.gov database as separate research questions (NCT00549172 and NCT01052233).
2) Accordingly, we have enrolled patients that represented optimal responders to this index surgical procedure. Also, surgeons carrying out the surgeries were highly experienced.
3) Conceding that the act of surgery *per se* produces a profound placebo response, a 'true' treatment effect is impossible to disentangle from the nonspecific (placebo or meaning) effects – such as the patients' or researchers' expectations of benefit – without a placebo comparison group.
4) The only difference between APM and placebo-surgery treatment groups is that the removal of torn segments of medial meniscus (partial meniscectomy) – the critical therapeutic (surgical) element – has been carried out for patients in the APM group.
    a. The critical therapeutic (surgical) element is the component of the surgical procedure that is believed to provide the therapeutic effect (here, APM), being distinct from aspects of the procedures that are diagnostic or required to access the disease being treated (here, knee arthroscopy).

Sihvonen R, *et al. Br J Sports Med* 2020; 54:1332–1339. doi: 10.1136/bjsports-2020-102813

   b. Apart from the critical therapeutic element, the treatment of the APM and placebo-surgery groups is identical, i.e., all "placebo or meaning effect" related to the entire treatment and care is identical.

5) Considering the above noted, to be deemed beneficial, APM has to be superior to placebo-surgery in one of the following two objectives and not inferior in the other, i.e.:
   a. APM should provide a statistically significant benefit over Placebo-surgery in at least one of the three primary outcomes and not to be inferior in the other two (NCT00549172).
   b. APM should provide a benefit (delay the development/progression of) in radiographic knee OA (NCT01052233), either by showing:
      i. A lower proportion of patients with development/progression of radiographic knee OA (development/progression defined as an increase of one grade or more in the Kellgren-Lawrence (KL) knee OA grading) or
      ii. A lower OARSI sum score (OARSI sum score is defined as the sum of marginal tibiofemoral osteophyte grades and tibiofemoral joint space narrowing (JSN) grades).

   Given that the study was not powered for the latter objective (NCT01052233), the interpretation will be based on the effect sizes included in the 95% confidence intervals.

In the scenario that APM proves beneficial in either of the two objectives, but inferior in the other, then we will proceed into careful consideration of the benefit/harm -ratio based on effect sizes.

Accordingly, our interpretation scheme is as follows:

	Beneficial			No difference	Harmful			Inconclusive	
**PROMS**	+	+	0	0	-	-	0	+	-
**Radiographic OA**	+	0	+	0	-	0	-	-	+

+ denotes Benefit of APM; 0 denotes No difference; - denotes Harm of APM (relative to placebo-surgery)

We will also perform sensitivity analysis taking into account unblindings/surgeries carried out during the 5-year follow-up with particular emphasis on the high tibial osteotomies/knee arthroplasties (as they may have a relevant effect on the PROMs).

Statistical commitments for main interpretation:

a) I-T-T is the primary data analysis for both objectives, but sensitivity analysis will also be carried out in the case of "**Inconclusive**" findings.
b) The prespecified time point of primary interest is 5 years after arthroscopy.

FIDELITY Finnish Degenerative Meniscus Lesion Study

FIDELITY Trial: Minutes of the Blinded Data Interpretation meeting of the 5-yr follow-up

Lund, Sweden June 26, 2019 at 11:45.

Aleksandra Turkiewicz, chair

Raine Sihvonen

Pirjo Toivonen

Martin Englund

Simo Taimela

Teppo Järvinen, secretary

Velocity Hughes, External observer

3

FIDELITY Finnish Degenerative Meniscus Lesion Study

FIDELITY Trial: Minutes of the Blinded Data Interpretation meeting of the 5-yr follow-up

MINUTES OF THE "BLINDED REVIEW OF THE DATA"

The Writing committee of the FIDELITY trial (undersigned, below) developed and recorded two interpretations of the results on the basis of a blinded review of the primary outcome data (treatment A compared with treatment B), with one assuming that A was the Arthroscopic partial meniscectomy (APM) group and another assuming that A was the Placebo-surgery group.

The meeting started on June 26, 2019, at 12:00. All Writing Committee members were present and statistician Aleksandra Turkiewicz (AT) began to present the results. AT had coded the Groups as Group A and Group B and at this point, she was the only one aware of the allocation codes of the groups. Also, as Dr. Sihvonen and research coordinator Pirjo Toivonen had previous access to the data, they recused themselves from making any interpretations.

General principles for blinded results presentation

The current results are for 3- to 5-year follow-up. The results from 1- and 2-year follow-ups, including baseline data, have already been published and known. These data were repeated when appropriate without blinding. However, the longer term follow-up data (from 3 to 5 years) were presented in blinded manner and not linked to any baseline, 1- or 2-year data to prevent unblinding. This is why the detailed results (with estimated regression coefficients for all parameters) were not shown, as this would lead to unblinding.

Data Presented by the Statistician

The data shown in blinded data interpretation (AT's Powerpoint presentation) is attached as an appendix to this document.

Primary Comparison

Objective 1: Efficacy of APM (NCT00549172):

While reviewing the blinded results, the undersigned noted that there is no statistically significant (nor clinically relevant) difference in any of the three primary outcomes (Appendix, Table 6, below).

Sihvonen R, *et al. Br J Sports Med* 2020; 54:1332–1339. doi: 10.1136/bjsports-2020-102813

FIDELITY Finnish Degenerative Meniscus Lesion Study

FIDELITY Trial: Minutes of the Blinded Data Interpretation meeting of the 5-yr follow-up

Table 6. Results from statistical analyses – primary objective 1.

Outcome	The *difference* between the groups (95%CI) at 5 years
WOMET	1.7 (-4.3, 7.7)
Lysholm knee score	2.1 (-2.6, 6.8)
Knee pain after exercise	.04 (-0.72, 0.81)

Objective 2: Development/progression of radiographic knee OA (NCT01052233):

While reviewing the blinded results, the undersigned noted that there is a consistent, slightly greater development/progression of radiographic OA in Group A (Appendix, Table 7, below).

Table 7. Results from statistical analyses – primary objective 2.

**Outcome**	**The *risk* difference between the groups (95%CI) at 5 years**
Radiographic *development/progression* of OA, 1 grade	-0.11 (-0.26, 0.05)
**Outcome**	**The *difference in score* between the groups (95%CI) at 5 years**
Sum of OARSI grades	-1.0 (-1.8, -0.3)

Three persons who had a total knee replacement (TKR) during the follow-up had their radiological scores at 5 years set to KL grade 4 and OARSI sum score to 12. One person who had a high tibial osteotomy (HTO) had his/her radiological score at 5 years set to KL grade 3 and OARSI sum score to 9. These scores for TKR and HTO were pre-defined. As this can have an effect on both primary analyses (both PROMs and radiographical outcomes), we decided to carry out a sensitivity analysis to test the robustness of our findings.

Sensitivity analyses

The exclusion of 4 people with TKR/HTO did not materially change the results (below).

FIDELITY Trial: Minutes of the Blinded Data Interpretation meeting of the 5-yr follow-up

Primary objective #1, included 142 persons

Outcome	The *difference* between the groups (95%CI) at 5 years
WOMET	1.8 (-4.1, 7.7)
Lysholm knee score	2.3 (-2.4, 7.0)
Knee pain after exercise	-0.01 (-0.79, 0.76)

Primary objective #2, included 137 persons with non-missing structural data

Outcome	The *risk* difference between the groups (95%CI) at 5 years
Radiographic *development/progression* of OA, 1 grade	-0.10 (-0.26, 0.06)
**Outcome**	**The *difference in score* between the groups (95%CI) at 5 years**
Sum of OARSI grades	-0.7 (-1.4, -0.1)

Primary Interpretation of the Results

Accordingly, depending on group assignment, our findings suggest one of the two scenarios: APM does not have a beneficial effect on knee symptoms or function, but either 1) slightly accelerates or 2) slightly delays the development/progression of radiographic OA.

	Beneficial	Harmful
PROMS	0	0
Radiographic OA	+	-

+ denotes Benefit of APM; 0 denotes No difference; - denotes Harm of APM (relative to placebo-surgery)

Given the relatively small effect sizes and the uncertainty of our point estimates for the objective #2 (NCT01052233), our clinical interpretations will be primarily based on patient-relevant outcomes.

Clinical implications of the findings

a) If Group A = APM and Group B = Placebo-surgery

Our results suggest that patients with a degenerative medial meniscus tear do not benefit from APM compared to placebo-surgery with respect to patient-relevant outcomes (knee symptoms and function) at 5 years after surgery. Further, APM may be associated with a slightly greater development/progression of radiographic OA. Taken together, these findings further strengthen the latest clinical practice guidelines making a strong recommendation against the use of APM in nearly all patients with a degenerative meniscus tear.

Sihvonen R, *et al. Br J Sports Med* 2020; 54:1332–1339. doi: 10.1136/bjsports-2020-102813

b) If Group A = Placebo-surgery and Group B = APM

Our results suggest that patients with a degenerative medial meniscus tear do not benefit from APM compared to placebo-surgery with respect to patient-relevant outcomes (knee symptoms and function) at 5 years after surgery. However, APM may be associated with a slightly lesser development/progression of radiographic OA. Given that no benefit of APM was seen in the patient-relevant outcomes, we feel that our findings do not warrant a change in the latest clinical practice guidelines. However, these findings call for further studies on the possible protective effect of APM on the progression of OA and its potential mechanisms.

Analysis of the Secondary Outcomes

Given the hypothesis-generating/explanatory nature of the secondary outcomes, they were not interpreted.

Unblinding the group assignment

At 15:25 the randomization code was broken, revealing that Group A was APM and Group B was Placebo-surgery.

FINAL INTERPRETATION

Blinded data interpretation was conducted as planned.

Our results suggest that patients with a degenerative medial meniscus tear do not benefit from APM compared to placebo-surgery with respect to patient-relevant outcomes (knee symptoms and function) at 5 years after surgery. Further, APM may be associated with a slightly greater development/progression of radiographic OA. Taken together, these findings further strengthen the latest clinical practice guidelines making a strong recommendation against the use of APM in nearly all patients with a degenerative meniscus tear.

FIDELITY Finnish Degenerative Meniscus Lesion Study

FIDELITY Trial: Minutes of the Blinded Data Interpretation meeting of the 5-yr follow-up

We hereby confirm that this document describes the course of events truthfully during the blinded data interpretation meeting of the FIDELITY trial.

Lund, June 26, 2019, at 15:32.

Aleksandra Turkiewicz, chair

Raine Sihvonen

Pirjo Toivonen

Martin Englund

Simo Taimela

Teppo Järvinen, secretary

Appendices

1. Aleksandra Turkiewicz's Powerpoint presentation entitled: "FIDELITY trial 5-year follow-up assessments - blinded results"

8

15.4.2020

FIDELITY trial
5-year follow-up assessments
# - blinded results

1

Outline

- General considerations
- Descriptive data at baseline
- Descriptive data and inferential results for primary objective #1
- Descriptive data and inferential results for primary objective #2
- Descriptive data and inferential results for other outcomes

2

General principles for blinded results presentation

The current results are for 3- to 5-year follow-up. The results from 1- and 2-year follow-ups, including baseline data, are already published and known. These data will be repeated when appropriate without blinding. However, the (3-, 4- and) 5-year outcomes will be presented in blinded manner and also not linked to any baseline, 1- or 2-year data to prevent unblinding. This is why the detailed results (with estimated regression coefficients for all parameters) are not shown, as this would lead to unblinding. The arms in the blinded results are named “Group A” and “Group B”.

3

Data sources

All the analyses are based on data/variables as listed in the document:
FIDELITY_5y_data_overview_20190619.docx

4

Analysis plan

Analyses are based on analysis plan:

SAP-FIDELITY 5-yr_200619_ME_AT.doc

5

Interpretation of the inferential results

The same arm (denoted as “Group A”) is used as reference group in all analyses. Interpretation of the results should be made in light of values included in the respective 95% CIs. For example, I suggest that a conclusion of “no difference” is made only when a 95% CI excludes any clinically relevant difference. If a 95% CI includes both small irrelevant differences and large, potentially clinically important differences, then the results may be inconclusive.

6

Descriptive data, baseline

Table 1A. Characteristics at baseline (already known from previous publications):

Characteristic	APM	Sham
Age (years), mean(SD)	52 (7.2)	52.1 (6.9)
Sex, n(%)	47 (60)	42 (60)
KL grade 1*, baseline, n(%)	40 (50)	35 (50)
Tampere, n(%)	41 (54)	39 (56)
Helsinki, n(%)	6 (8)	3 (4)
Turku, n(%)	9 (12)	8 (11)
Jyväskylä, n(%)	6 (8)	8 (11)
Kuopio, n(%)	14 (18)	12 (17)
Weight (kg), mean(SD)	83.2 (14.6)	80.7 (14)
Height (m), mean(SD)	172.6 (9.3)	173.1 (8.4)
BMI, mean(SD)	27.9 (4)	26.9 (4)
WOMET, mean(SD)	52.8 (18.1)	56.4 (17.3)
Lysholm score, mean(SD)	60.1 (14.6)	60.2 (14.7)
Pain after exercise, mean(SD)	6.1 (2)	5.8 (2)

*according to original reading used for randomization purposes

7

OARSI sum score

- Joint space narrowing, medial: 0-3
- Osteophytes, femur, medial: 0-3
- Osteophytes, tibia, medial: 0-3

- Joint space narrowing, lateral: 0-3
- Osteophytes, femur, lateral: 0-3
- Osteophytes, tibia, lateral: 0-3

- Sum score: 0-18

8

Descriptive data, baseline

Table 1B. Characteristics at baseline. KL grade (according to new readings) and OARSI sum score.

	Group A	Group B
KL grade, (%)		
0	43	46
1	30	20
2	23	33
3	3	1
OARSI sum score, mean (SD)	1.3 (1.5)	1.1 (1.2)

9

Follow-up data, overview

Table 2. Missing data.

Follow-up timepoints	Number of non-missing values					
	WOMET	Lysholm	Pain after exercise	K-L grades	OARSI grades	Clinical OA according to ACR criteria
Baseline	146	146	146	145	145	Not applicable
6 months	146	145	146			
12 months	146	145	146			
24 months	144	143	144			
36 months	141	138	140			
48 months	143	143	143			
60 months	142	141	142	141	141	137

10

Follow-up data, primary objectives #1

Table 3. Descriptive data on the study outcomes, PROM.

Time point	WOMET, mean (SD)	Lysholm score, mean (SD)	Pain after exercise, mean (SD)
Baseline	54.5 (17,8)	60.2 (14.6)	6.0 (2.0)
2 months	73.1 (21.1)	76.6 (16.3)	3.6 (2.5)
6 months	80.4 (20.7)	82.7 (15.0)	2.8 (2.4)
12 months	80.4 (20.8)	82.8 (14.6)	2.8 (2.5)
24 months	83.8 (18.3)	84.7 (14.3)	2.3 (2.5)
36 months	83.3 (18.9)	84.3 (14.5)	2.1 (2.3)
48 months	84.1 (18.3)	84.5 (15.2)	2.2 (2.4)
60 months	84.6 (18.7)	84.8 (15.8)	2.1 (2.5)

11

Follow-up data, primary objectives #1

Table 4. Descriptive data on the study outcomes, 3 to 5 years, per group.

	WOMET, mean (SD)		Lysholm score, mean (SD)		Pain after exercise, mean (SD)	
	**Group A**	**Group B**	**Group A**	**Group B**	**Group A**	**Group B**
**Time point**						
36 months	83.9 (17.7)	82.7 (20.2)	84.5 (15.4)	84.1 (13.6)	1.8 (2.2)	2.3 (2.4)
48 months	83.2 (19.8)	85.0 (16.9)	83.0 (17.1)	85.9 (13.3)	2.4 (2.5)	2,0 (2.2)
60 months	84.5 (18.5)	84.6 (19)	83.8 (17.2)	85.7 (14.5)	2.0 (2.5)	2.2 (2.4)

12

15.4.2020

Analysis results, primary objective #1

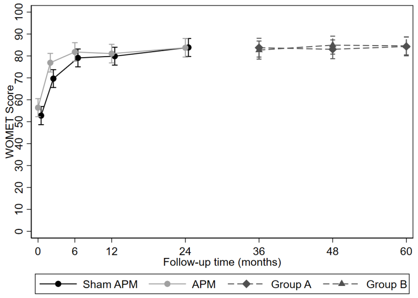

13

Analysis results, primary objective #1

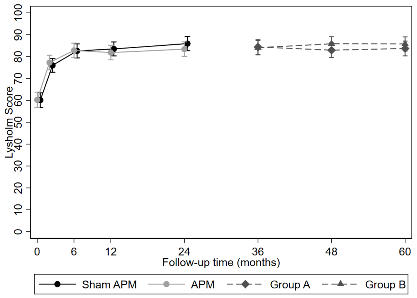

14

Analysis results, primary objective #1

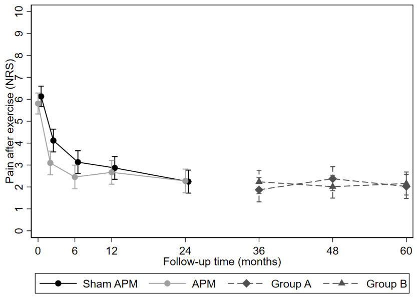

15

Analysis results, primary objective #1

Table 6. Results from statistical analyses – primary objective 1.

Outcome	The *difference* between the groups (95%CI) at 5 years
WOMET	1.7 (-4.3, 7.7)
Lysholm knee score	2.1 (-2.6, 6.8)
Knee pain after exercise	.04 (-0.72, 0.81)

16

BMJ Publishing Group Limited (BMJ) disclaims all liability and responsibility arising from any reliance placed on this supplemental material which has been supplied by the author(s)

15.4.2020

Follow-up data, primary objective #2

Table 5. Summary of radiographic outcomes at 5 years (number of knee replacements presented only for all cohort to prevent potential unblinding):

	All		Group A	Group B
**Outcome**	**N**	**%**	**%**	**%**
Progression in KL grades (at least 1 grade)	87	62	67	57
Progression in KL grades (at least 0.5 grade)	109	77	81	75
Clinical knee OA according to ACR criteria	11	8	8	9
Number of knee replacements or osteotomies	4	3		
	**Mean (SD)**		**Mean (SD)**	**Mean (SD)**
OARSI sum score*	3.2 (3.0)		4.0 (3.3)	2.8 (2.4)

*Three persons had knee replacement during follow-up, their radiological scores at 5 years were set to KL grade 4 and OARSI sum score 12. One person had osteotomy, this person's radiological scores at 5 years were set to KL grade 3 and OARSI sum score 9.

17

Follow-up data, primary objective #2

Figure 1. Overview of the *difference* in OARSI scores between 5 years and baseline, histogram.

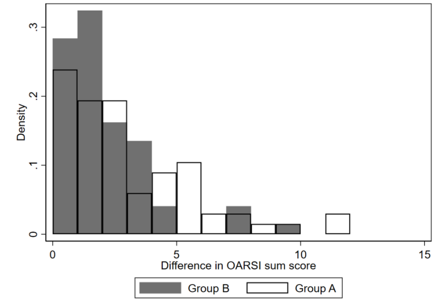

18

Analysis results, primary objective #2

The results for *binary* outcomes are presented as risk differences, i.e. the difference between the groups in the proportion of persons with a given outcome at 5 years. For example, a difference of 0.1 means that the proportion of persons with an outcome in one group is 10% larger than in the other group (for example 30% and 20%, or 60% and 50%, respectively).

19

Analysis results, primary objective #2

Table 7. Results from statistical analyses – primary objective 2.

Outcome	The *risk* difference between the groups (95%CI) at 5 years
Radiographic *progression* of OA, 1 grade	-0.1 (-0.26, 0.05)
Radiographic *progression* of OA, 0.5 grade	-0.07 (-0.20, 0.07)
Knee OA according to ACR criteria	0.01 (-0.08, 0.09)
KR or osteotomy	Only descriptive due to few events, see above for summary for the whole study sample
Outcome	The *difference in score* between the groups (95%CI) at 5 years
Sum of OARSI grades	-1.0 (-1.8, -0.3)

20
