# Supplementary material for: Arthroscopic partial meniscectomy for a degenerative meniscus tear: a 5 year follow-up of the placebo-surgery controlled FIDELITY (Finnish Degenerative Meniscus Lesion Study) trial
Source: Br J Sports Med. 2020 Aug 27;54(22):1332–9. doi: 10.1136/bjsports-2020-102813 (PMC7606577; doi:10.1136/bjsports-2020-102813)
Supplement: Supplementary data [file bjsports-2020-102813supp002.pdf]

Appendix 2. Reliability of radiographic assessment of knee osteoarthritis

A. Comparison of radiological readings between a consensus of two readers (consensus) and an independent, third reader (single reader).

Outcome	CONSENSUS	SINGLE READER
Radiographic *progression* of OA, 1 grade*	-0.13 (-0.28, 0.02)	-0.11 (-0.26, 0.05)
Radiographic *progression* of OA, 0.5 grade*	-0.14 (-0.26, -0.03)	-0.07 (-0.20, 0.07)
Sum of OARSI grades§	-0.7 (-1.3, -0.1)	-1.0 (-1.8, -0.3)

* The *risk difference* between the Sham APM and APM arm (95%CI) at 5 years
§ The *difference* between the Sham APM and APM (95%CI) at 5 years

B. Agreement of Kellgren-Lawrence (KL) and OARSI grading by a consensus reading of two new readers vs. a single reader.

Please see the methods section for details on the procedure of radiographic readings.

We estimated the agreement of KL baseline scores, KL scores at 5 years and progression of at least 1 grade (primary outcome) and of at least 0.5 grades (outcome for sensitivity analysis) as well as difference in OARSI grades between 5 years and baseline (primary outcome).

For KL grades (ordinal data) we used weighted kappa with weights of the form 1-|i-j|/4 weights (i and j are rows and columns of the ratings). The progression of KL grades (binary outcome) we used unweighted kappa. The CIs for kappa are bias-corrected from 1000 bootstrap replicates.

For OARSI scores we estimated the intraclass correlation (ICC) treating the persons are random and readers as fixed, absolute agreement is reported.

Outcome	Consensus vs. Single reader
KL grades at baseline, weighted kappa (95%CI)	0.53 (0.43 - 0.63)
KL grade at 5y follow-up, weighted kappa (95%CI)	0.58 (0.49 - 0.68)
KL progression with at least 1 grade, kappa (95%CI)	0.27 (0.11 – 0.44)
KL progression with at least 0.5 grade, kappa (95%CI)	0.44 (0.24 – 0.60)
Difference in Sum OARSI score 5y-baseline, ICC (95%CI)	0.77 (0.69 – 0.83)
