# Supplementary material for: Arthroscopic partial meniscectomy for a degenerative meniscus tear: a 5 year follow-up of the placebo-surgery controlled FIDELITY (Finnish Degenerative Meniscus Lesion Study) trial
Source: Br J Sports Med. 2020 Aug 27;54(22):1332–9. doi: 10.1136/bjsports-2020-102813 (PMC7606577; doi:10.1136/bjsports-2020-102813)
Supplement: Supplementary data [file bjsports-2020-102813supp004.pdf]

Sihvonen *et al. Trials* (2020) 21:76
https://doi.org/10.1186/s13063-019-3833-2

Trials

STUDY PROTOCOL Open Access

Statistical analysis plan for the 5-year and 10-year follow-up assessments of the FIDELITY trial

Raine Sihvonen[1], Roope Kalske[2], Martin Englund[3,4], Aleksandra Turkiewicz[3], Pirjo Toivonen[2], Simo Taimela[2], Teppo L. N. Järvinen[2*] and for the Finnish Degenerative Meniscal Lesion Study (FIDELITY) Investigators

Abstract

Background: The research objectives of the 5-year and 10-year assessments in the Finnish degenerative meniscal lesion study (FIDELITY) are twofold: (1) to assess the long-term efficacy of arthroscopic partial meniscectomy (APM) in adults (age 35 to 65 years) with a degenerative meniscus tear and (2) to determine the respective effects of APM and degenerative meniscus tear on the development of radiographic and clinical knee osteoarthritis (OA).

Methods and design: FIDELITY is an ongoing multi-center, randomized, participant and outcome assessor blinded, placebo-surgery-controlled trial in 146 patients. This statistical analysis plan (SAP) article describes the overall principles for analysis of long-term outcomes (5-year and 10-year follow up), including how participants will be included in each analysis, the primary and secondary outcomes and their respective analyses, adjustments for covariates, and the presentation of the results. In addition, we will present the planned sensitivity and subgroup analyses.

Discussion: To assess the long-term efficacy of APM on knee symptoms and function we are carrying out a long-term (5-year and 10-year) follow up of our placebo-surgery-controlled FIDELITY trial according to statistical principles outlined in detail in this document. As our second primary objective, whether APM (resection of torn meniscus tear) accelerates or delays the development of knee osteoarthritis in patients with an arthroscopically verified degenerative tear of the medial meniscus, a pre-registered follow-up is also carried out.

Trial registration: ClinicalTrials.gov, NCT00549172 (Arthroscopy in the Treatment of Degenerative Medial Meniscus Tear). Registered on 25 October 2007 (NCT00549172). ClinicalTrials.gov, NCT01052233 (Development of Knee Osteoarthritis After Arthroscopic Partial Resection of Degenerative Meniscus Tear). Registered on 20 January 2010.

Keywords: Statistical analysis plan, Randomized controlled trial, Arthroscopic partial meniscectomy, Osteoarthritis

Introduction

Trial overview and purpose of the statistical analysis plan

The Finnish degenerative meniscal lesion study (FIDELITY) is a trial to assess the efficacy of arthroscopic partial meniscectomy (APM) in patients with a degenerative meniscus tear. The primary outcome assessment point of the trial was at 1 year post surgery. The original study protocol [1] and the results of 1-year and 2-year analyses [2, 3] and a secondary analysis focusing on mechanical symptoms [4] are published.

To safeguard against the imminent risk of outcome reporting bias, selective reporting, and data-driven interpretation of results, this statistical analysis plan (SAP, Version 2.0) for the 5-year and 10-year follow up is published as an update to the previously published protocol [1]. The original study protocol [1] provides more details on the trial rationale, eligibility criteria, interventions, data management, and methods for limiting bias. This SAP follows the guidelines for writing SAPs provided by Gamble et al. [5] and describes the overall principles for analysis of long-term outcomes (5-year and 10-year

* Correspondence: teppo.jarvinen@helsinki.fi
[2]Finnish Center for Evidence-Based Orthopedics (FICEBO), Department of Orthopedics and Traumatology, University of Helsinki and Helsinki University Hospital, Töölö hospital, Topeliuksenkatu 5, Building B, 00260 Helsinki, Finland
Full list of author information is available at the end of the article

© The Author(s). 2020 **Open Access** This article is distributed under the terms of the Creative Commons Attribution 4.0 International License (http://creativecommons.org/licenses/by/4.0/), which permits unrestricted use, distribution, and reproduction in any medium, provided you give appropriate credit to the original author(s) and the source, provide a link to the Creative Commons license, and indicate if changes were made. The Creative Commons Public Domain Dedication waiver (http://creativecommons.org/publicdomain/zero/1.0/) applies to the data made available in this article, unless otherwise stated.

follow up), including how participants will be included in each analysis, the primary and secondary outcomes and their respective analyses, adjustments for covariates, and the presentation of the results. In addition, we will present the planned sensitivity and subgroup analyses. The trial results will be reported according to the Consolidated standards of reporting trials (CONSORT) guidelines for randomized controlled trials (RCTs) [6].

Background

By the end of the 21st century, APM had become the most common orthopedic procedure with well over half a million such surgeries performed annually in the USA alone [7, 8], mostly in middle-aged and older patients [8]. According to conventional wisdom, APM was thought to result in short-term improvement in knee function and quality of life. However, a series of rigorous trials, summarized in three recent systematic reviews and meta-analyses, provide compelling evidence that APM offers little short-term to medium-term benefit above sham surgery or non-surgical management in most patients with knee pain and degenerative meniscus tear [9, 10]. Recent evidence thus convincingly contradicts the widely held contention that APM is beneficial in improving knee symptoms or function, but there is still uncertainty about the possible undesirable consequences of the procedure [11]. Overall, the risk of adverse events within 90 days of the procedure appears low, but serious adverse events (including pulmonary embolism and infection) have been associated with this surgery [12, 13].

However, there is mounting evidence to suggest that APM is associated with increased risk of accelerated progression of knee osteoarthritis (OA) and earlier need for "corrective" surgery (high tibial osteotomy (HTO) or total knee replacement (TKR)) in middle-aged to older patients [14, 15]. It still remains unclear whether the increased risk is due to the meniscus tear per se, the surgical procedure (APM), or if there is an interaction between the two. This question cannot be addressed simply by evaluating the outcome of patients who have undergone APM, because the role of the underlying degenerative process and the surgical procedure cannot be disentangled in such a design [16]. Given the current uncertainty about the potential effect of APM on the development or progression of knee OA, we are planning to address this particular issue by carrying out an adjunct, pre-registered analysis of the FIDELITY trial at 5 and 10 years after randomization. The biological rationale behind these studies is that resection of the torn meniscus (APM) has an effect on the progression of degenerative knee disease: some argue that APM cures symptoms and slows down the development of OA while others assert the contrary.

Objectives

The following two research questions capture the primary objectives of these 5-year and 10-year follow-up investigations:

1. What is the long-term efficacy of APM (versus placebo surgery) on functional outcome and knee symptoms in patients with an arthroscopically verified degenerative tear of the medial meniscus?
2. Does APM either accelerate or delay the development/progression of radiographic and clinical knee OA in these patients?

Trial design

FIDELITY is a multicenter, randomized, participant and outcome assessor blinded, placebo-surgery-controlled trial. This study is carried out at five orthopedic clinics of Tampere University Hospital Hatanpää, Kuopio University Hospital, Helsinki Central Hospital (Jorvi), Turku University Hospital, and the Central Finland Central Hospital in Jyväskylä, all in Finland. The study group at each center consists of a main investigator (an orthopedic surgeon experienced in knee arthroscopy) who took care of the recruitment of the patients and all surgical procedures, a study nurse, an orthopedic surgeon for possible postoperative problems and another for scheduled follow-up examinations, the latter two both blinded to the treatment allocation. Patients were enrolled between 2007 and 2012 and all follow-up assessments were carried out between October 2013 and January 2017. The study was approved by the Pirkanmaa Hospital District's committee of ethics (no. R06157). The two research questions were registered as separate studies in the ClinicalTrials database (Clinicaltrials.gov identifiers NCT00549172 and NCT01052233). The study process shown in Fig. 1 provides a brief outline of the trial. The eligibility criteria for the study are presented in Box 1.

Methods

Outcomes

Objective 1: efficacy of arthroscopic partial meniscectomy (NCT00549172)

To assess the efficacy of APM (versus placebo surgery) on the functional outcome and knee symptoms in patients with an arthroscopically verified degenerative tear of the medial meniscus, we will be using the same three patient-relevant outcomes (PROMs) that were used as our primary outcomes in the previous, 1-year and 2-year follow-up publications of this data [2, 3].

The primary outcomes are:

1. Western Ontario Meniscal Evaluation Tool (WOMET) score, a disease-specific instrument for assessing quality of life that was developed and

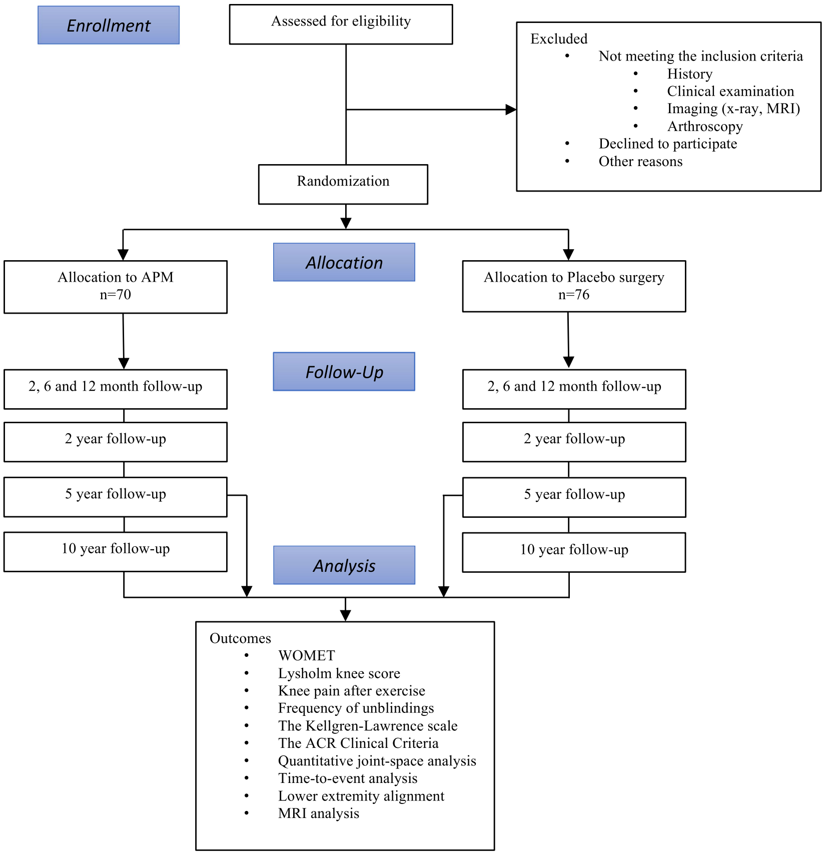

Fig. 1 An outline of the study process of the FIDELITY trial

validated for patients with meniscal pathologic conditions [17, 18]
2. Lysholm knee score, the most commonly used outcome instrument for various knee conditions [19, 20] and a tool that has also been validated for patients with meniscal injury [21]
3. Knee pain after exercise, assessed on an 11-point scale ranging from 0 (no pain) to 10 (extreme pain)

The secondary outcome is the frequency of unblinding in the two study groups: patients with inadequate relief of symptoms underwent unblinding of the treatment-group allocation.

These outcomes and the justification for these have previously been elaborated in detail [1–4].

Objective 2: development of knee OA (NCT01052233)

To assess whether APM either accelerates or delays the development/progression of radiographic and clinical knee OA in these patients, we will use radiographs and established clinical criteria to assess the progression of knee OA at the 5-year and 10-year time point after the index surgeries as follows.

The primary outcomes are:

1. Development/progression of radiographic OA
   a. An increase of one grade or more in the Kellgren-Lawrence (KL) knee OA grading (dichotomous outcome: yes or no)
   b. The KL scale is a semi-quantitative instrument (ordered categorical grades 0–4) to assess the severity of radiographic tibiofemoral knee OA [22]. Patients who have undergone an osteotomy or a total knee replacement during follow up will be considered to have progressed radiographically according to the definition above.
2. Radiographic progression based on the sum of marginal tibiofemoral osteophyte grades and tibiofemoral joint space narrowing (JSN) grades (Osteoarthritis Research Society International (OARSI)) atlas (continuous outcome, hypothetical range 0–18)

Box 1 Inclusion and exclusion criteria used in the FIDELITY trial

Inclusion criteria

Age: 35–65 years of age

Persistent (> 3 months) pain on the medial joint line of the knee

Pain provoked by palpation or compression of the joint line or a positive McMurray sign

MRI showing signals characteristic of medial meniscus injury

Degenerative injury to the medial meniscus confirmed at arthroscopy

Exclusion criteria

Trauma-induced onset of symptoms

Locked knee (that cannot be straightened normally)

Previous surgical procedure on the affected knee

Clinical osteoarthritis (OA) of the knee (American College of Rheumatology criteria)

Radiological OA of the knee (Kellgren-Lawrence grade > 1) at clinical site readings

Acute (within the previous year) fracture of the affected extremity

Decreased range of motion of the knee

Instability of the knee

MRI assessment shows pathology other than degenerative knee disease requiring treatment other than arthroscopic partial meniscectomy (APM)

Arthroscopic examination reveals pathologic change other than a degenerative injury to the medial meniscus requiring intervention other than APM

MRI magnetic resonance imaging, *OA* osteoarthritis

The OARSI atlas is a semi-quantitative instrument (ordered categorical grade 0–3) with focus to assess the severity of JSN and osteophytes, respectively, in knee OA [23].

The secondary outcome:

1. Knee OA according to the American College of Rheumatology Clinical Criteria [24]

Auxiliary outcomes:

2. Development/progression of radiographic OA by a grade increase of 0.5 or more in the Kellgren-Lawrence (KL) knee OA grading (dichotomous outcome: yes or no)
   a. More sensitive than a one full grade (above) but may potentially capture "too many" patients as progressed in the two treatment arms, in particular at the 10-year follow up
3. Quantitative analysis of the joint-space width based on radiographs
4. Time-to-event analysis (OA-related surgery, arthroplasties or osteotomies)
5. Magnetic resonance imaging (MRI)-based progression by semi-quantitative scoring (MOAKS) [25]
6. MRI-based progression of knee degeneration by quantitative assessment of change in OA features (cartilage, bone, bone marrow lesions, synovitis, and meniscus integrity and extrusion)
7. Lower extremity alignment (mechanical axis): change from baseline to 5 years
8. Patient satisfaction and self-rated improvement
9. Patients' return to normal activities
10. The presence of mechanical symptoms [4]
11. Clinical knee examination
12. Serious adverse events
13. Frequency of repeat APM, and the number of osteotomies and knee arthroplasties
14. Possible derivates from the above noted outcomes

For all radiographic outcomes, two experienced musculoskeletal radiologist (JK, NS), unaware of the treatment allocation and clinical data, will grade the baseline, and the 5- and 10-year radiographs of the operated (index) knee of all participants. After readings by both readers, a consensus will be sought. All the analyses of secondary outcomes are supportive, exploratory, and/or hypothesis-generating.

Rationale for outcomes to be reported and for the statistical analyses

For the assessment of the efficacy of APM (NCT00549172), we will use the same PROMs used in the previous publications depicting the 1-year [2] and 2-year [3] follow-up findings. To safeguard against potential multiplicity effects [26] in this analysis, we will interpret the treatment effect estimates and their 95% CIs for all our three primary outcomes.

As for the evaluation of the development/progression of knee OA (NCT01052233), the 5-year follow up is the first time point one can reasonably expect any OA-related changes to take place or to be quantifiable. Having said that, the outcome measures we originally registered in the ClinicalTrials.gov database (i.e., KL grade and OA as defined by the American College of Rheuatologists (ACR) clinical criteria) are quite insensitive to change, so we have decided to add the sum of OARSI atlas osteophyte grades and JSN grades as an additional primary outcome of radiographic progression of OA.

Statistical analysis

All the analyses will be performed according to the intention to treat (ITT) principle or, if impossible, using the full analysis set [27]. In sensitivity analysis we will also perform per-protocol (PP) analyses. For all outcomes, 95%

confidence intervals for the relevant between-group differences will be calculated.

Mixed model linear regression will be used to analyze continuous outcomes related to objective 1 (efficacy of APM). In this model the patient will be included as the random effect and time point (6, 12, 24, 60, or 120 months), treatment arm (APM or placebo) and their interaction, and randomization stratification factors, i.e., age (35–50 years or 51–65 years), sex, absence or presence of minor degenerative changes on a radiograph (KL grade 0 or 1), and study center, will be included as fixed effects. The model will be adjusted for baseline values of the respective outcome variable.

Logistic regression will be used to analyze the binary outcomes. The model will be adjusted for the baseline randomization stratification factors (age (35–50 years or 51–65 years), sex, and absence or presence of minor degenerative changes on a radiograph (KL grade 0 or 1). The method of standardization will be used to obtain the adjusted risk ratio and the adjusted risk difference from the logistic regression model [28]. Although randomization was also stratified by study site, we will *not* adjust for site in the logistic regression analysis due to the small number of participants in some centers and anticipated sparse data. Sensitivity will be analyzed including the study site as a covariate. We will use a linear regression model adjusted for randomization stratification variables and the baseline value of the outcome to analyze continuous outcomes related to objective 2. Serious adverse events will also be reported.

Study power considerations

We note that we originally powered the study to detect a minimal clinically important difference in the efficacy (patient-reported) outcomes - the Lysholm and WOMET scores (differences of at least 11.5 and 15.5 points, respectively) and in the score for knee pain after exercise (difference of at least 2.0 points) - between the APM and placebo-surgery groups. The original sample size calculation for the FIDELITY trial - which was geared at assessing the (short-term) efficacy of APM on pain and function - yielded a sample size of 70 patients per group. At the moment, having completed the 5-year follow up, we know that we have 96% adherence to follow up (68/70 patients in the APM and 72/76 in the placebo-surgery groups, respectively). Based on this, we anticipate the loss to follow up to be no more than 5 additional patients per group at the end of the follow-up period (at 10 years), thus leaving approximately 63–67 patients per group for the final analyses. These values provide us with 80% power, based on a two-sided type 1 error rate of 5%, to detect a 20% unit difference in the proportion of knee OA between APM and placebo-surgery. However, we consider it important to report estimates of the difference with a measure of uncertainty (such as 95% confidence intervals), even if smaller differences cannot be declared as statistically significant in a conventional way. This is due to two reasons: (1) even a statistically non-significant difference can potentially exclude a clinically relevant difference in one direction (e.g., with a 95% CI of – 3% to 15% for comparison between APM and sham APM in the frequency of knee OA at 5 years, we can exclude a clinically relevant difference favoring APM); (2) even if the results of this particular study are inconclusive, they can inform any future meta-analysis, as we can expect the estimate to be unbiased due to the stringent design of the study.

Blinded data interpretation

Given the widely acknowledged importance of adequate blinding of all stakeholders in eliminating potential bias from the findings of RCTs [29], we have decided to add another safeguard, a procedure we coined *blinded data interpretation* [30]. In brief, our trial statistician (AT) will perform all statistical analyses using unblinded treatment groups and then provides the Writing Committee of the trial with blinded results (groups labeled group A and group B). The Writing Committee then contemplates on the interpretation of the results until a consensus is reached and agrees in writing on all alternative interpretations of the findings. We record the minutes of this meeting in a document coined statement of interpretation, which will be signed by all members of the Writing Committee. Only after a common agreement is reached, will the data manager and the trial statistician break the randomization code and the correct interpretation be chosen. A draft manuscript will then be finalized. Detailed minutes of blinded-data-interpretation meetings will be provided as a supplement to the manuscript. As our co-Principal Investigator (PI) (RS), research coordinator (PT), and trial statistician (AT) have performed statistical analyses for the previous publications of this trial [2–4], they will abstain from taking an active role in the blinded-data-interpretation meeting.

Ethical considerations

The study was approved by the Pirkanmaa Hospital District committee of ethics (no. R06157). Our application contained a specific, 6-point ethical analysis focusing on the methodological rationale for use of placebo surgery, risk-benefit assessment, and informed consent (for detail, see [1]).

Dissemination

The findings of this study, whether positive, negative or neutral, will be disseminated widely through peer-reviewed publications and conference presentations.

Trial status

The enrollment for the study was carried out between December 2007 and January 2012, and subsequently, the follow-up examinations took place between December 2013 and January 2017. We have now completed the 5-year follow-up examinations and data management and are ready to carry out blinded data interpretation of the 5-year data. The 10-year follow-up examinations are ongoing.

Discussion

Arthroscopic partial meniscectomy (APM) to treat persistent knee pain in middle-aged and older patients is one of the most common orthopedic surgical procedures in use, despite mounting evidence of no or only marginal benefits on patient-relevant outcomes. A degenerative meniscal tear has been reported to be an independent risk factor for progression of cartilage damage and the subsequent development of knee OA. However, the respective roles and individual contributions of meniscal tear and APM in the progression of OA remain unclear. Current evidence, primarily based on observational data and unblinded randomized controlled trials, suggest that APM increases the risk of development of knee OA, but the studies are hampered by confounding by indication or high rates of crossover and loss to follow up.

Our multicenter, randomized, placebo-surgery-controlled FIDELITY trial that involves patients with an arthroscopically verified degenerative medial meniscus tear provides an exceptionally rigorous design to address the aforementioned questions, namely whether arthroscopic partial meniscectomy is associated with an increased risk of progression of radiographic knee OA and whether APM has any beneficial effect on knee pain or function or other symptoms.

Supplementary information

Supplementary information accompanies this paper at https://doi.org/10.1186/s13063-019-3833-2.

Additional file 1: SPIRIT 2013 Checklist: Recommended items to address in a clinical trial protocol and related documents*.

Acknowledgements
We thank the patients for their participation. We would like to thank research nurses Marja-Liisa Sutinen, Sari Karesvuori, Pekka Karppi, Saara-Maija Hinkkanen, Johanna Koivistoinen, Kirsi Saarioja, Elina Jalava, Ina Fagerlund, and Marketta Rautanen for their vital role in the implementation of the study.
FIDELITY Investigators
Department of Orthopedics and Traumatology, Tays Hatanpää, Tampere University Hospital, Tampere, Finland
Raine Sihvonen, MD, PhD
Kari Kanto, MD
Timo Järvelä, MD, PhD
Janne Lehtinen, MD, PhD
Outi Päiväniemi, MD, PhD
Marko Raivio, MD
Pirjo Toivonen, PT
Department of Orthopedics and Traumatology, University of Helsinki and Helsinki University Hospital, Helsinki, Finland
Mika Paavola, MD, PhD
Juha Kalske, MD
Anna Ikonen, MD
Janne Karhunen, MD
Roope Sarvilinna, MD, PhD
Sikri Tukiainen, MD
Ville-Valtteri Välimäki, MD, PhD
Roope Kalske, MD
Simo Taimela, MD, PhD
Teppo LN Järvinen, MD, PhD
Department of Orthopedics and Traumatology, Tampere University Hospital, Tampere, Finland
Tero Järvinen, MD, PhD
Department of Radiology, Tampere University Hospital, Tampere, Finland
Niko Sillanpää, MD, PhD
Center for Health and Social Economics, National Institute for Health and Welfare, Helsinki, Finland
Antti Malmivaara, MD, PhD
Department of Orthopedics and Traumatology, Turku University Hospital, Turku, Finland
Ari Itälä, MD, PhD
Jani Knifsund, MD
Ville Äärimaa, MD, PhD
Department of Orthopedics and Traumatology, Kuopio University Hospital, Kuopio, Finland
Antti Joukainen, MD, PhD
Tommi Kääriäinen, MD
Heikki Kröger, MD, PhD, Professor
Janne Sahlman, MD
Department of Orthopedics and Traumatology, Central Finland Central Hospital, Jyväskylä, Finland
Heikki Nurmi, MD
Jukka Nyrhinen, MD
Juha Paloneva, MD, PhD
Department of Radiology, University of Tartu, Tartu, Estonia
Jaanika Kumm, MD, PhD
Clinical Epidemiology Unit, Orthopedics, Department of Clinical Sciences, Lund University, Lund, Sweden
Martin Englund, MD, PhD
Aleksandra Turkiewicz, MSc
Writing committee: R. Sihvonen, R. Kalske, S. Taimela, P. Toivonen, A. Turkiewicz, M. Englund, and T.L.N. Järvinen.
Trial concept and design: R. Sihvonen, A. Malmivaara, M. Paavola and T.L.N. Järvinen.
Provision of study materials or patients: T.L.N. Järvinen.
Statistical and epidemiological expertise: A. Turkiewicz and M. Englund
Obtaining of funding: T.L.N. Järvinen.
Administrative, technical, or logistic support: T.L.N. Järvinen and S. Taimela.
Patient enrollment: R. Sihvonen, A. Itälä, A. Joukainen, H. Nurmi, J. Kalske and J. Karhunen.
Acquisition, collection and assembly of data, responsibility for participants' follow-up:
2-year follow-up: T. Järvelä, T.A.H. Järvinen, J. Lehtinen, O. Päiväniemi, and M. Raivio (Hatanpää Hospital, Tampere, Finland); J. Nyrhinen and J. Paloneva, (Central Finland Central Hospital, Jyväskylä, Finland); S. Tukiainen and A. Ikonen (Helsinki University Central Hospital, Helsinki, Finland); H. Kröger and J. Sahlman (Kuopio University Hospital, Kuopio, Finland); J. Knifsund and V. Äärimaa (Turku University Hospital, Turku, Finland), and P. Toivonen (all sites).
5-year follow–up: K. Kanto (Hatanpää Hospital), T. Kääriäinen, (Kuopio University Hospital); V-V. Välimäki (Helsinki University Hospital); Ville Äärimaa, MD, PhD (Turku University Hospital), and P. Toivonen (all sites).
Radiographic readings: J. Kumm (Tartu University, Tartu, Estonia)
Research coordination: P. Toivonen.

Authors' contributions
All authors read and approved the final manuscript.

Funding
This study was supported by the Sigrid Juselius Foundation, the State funding for university-level health research (Tampere and Helsinki University Hospitals), the Social Insurance Institution of Finland (KELA), and the

Academy of Finland. Dr Sihvonen is supported also by the Finnish Medical Foundation and Orion-Pharmos Research Foundation. The funding sources had no role in the collection, analysis and interpretation of data; in the writing of the report and in the decision to submit the article for publication.

Availability of data and materials
Given that the informed consent forms of the FIDELITY trial did not include a provision for data sharing (trial launched in 2007), the full dataset cannot be shared due to a potential breach of the Finnish Personal Data Act. Scientists with a specific question regarding the trial data are encouraged to contact the corresponding author (TLNJ).

Ethics approval and consent to participate
Central ethical approval was confirmed from the Pirkanmaa Hospital District's committee of ethics (reference approval no. R06157) and we did not begin recruiting at other centers in the trial until local ethical approval was obtained. Informed consent was obtained from all study participants at trial entry.

Consent for publication
Not applicable.

Competing interests
All authors have completed the ICMJE uniform disclosure form at www.icmje.org/coi_disclosure.pdf and declare no support from any organization for the submitted work; no financial relationships with any organizations that might have an interest in the submitted work in the previous three years; no other relationships or activities that could appear to have influenced the submitted work.

Author details
[1]Department of Orthopedics and Traumatology, Tampere University Hospital, Hatanpää, Tampere, Finland. [2]Finnish Center for Evidence-Based Orthopedics (FICEBO), Department of Orthopedics and Traumatology, University of Helsinki and Helsinki University Hospital, Töölö hospital, Topeliuksenkatu 5, Building B, 00260 Helsinki, Finland. [3]Clinical Epidemiology Unit, Orthopedics, Department of Clinical Sciences Lund, Lund University, Lund, Sweden. [4]Clinical Epidemiology Research and Training Unit, Boston University School of Medicine, Boston, MA, USA.

Received: 30 July 2019 Accepted: 22 October 2019
Published online: 14 January 2020

References
1. Sihvonen R, Paavola M, Malmivaara A, Jarvinen TL. Finnish degenerative meniscal lesion study (FIDELITY): a protocol for a randomised, placebo surgery controlled trial on the efficacy of arthroscopic partial meniscectomy for patients with degenerative meniscus injury with a novel 'RCT within-a-cohort' study design. BMJ Open. 2013;3(3):e002510.
2. Sihvonen R, Paavola M, Malmivaara A, Itala A, Joukainen A, Nurmi H, Kalske J, Jarvinen TL. Arthroscopic partial meniscectomy versus sham surgery for a degenerative meniscal tear. N Engl J Med. 2013;369(26):2515–24.
3. Sihvonen R, Paavola M, Malmivaara A, Itala A, Joukainen A, Nurmi H, Kalske J, Ikonen A, Jarvela T, Jarvinen TAH, et al. Arthroscopic partial meniscectomy versus placebo surgery for a degenerative meniscus tear: a 2-year follow-up of the randomised controlled trial. Ann Rheum Dis. 2018;77(2):188–95.
4. Sihvonen R, Englund M, Turkiewicz A, Jarvinen TL. Mechanical symptoms and arthroscopic partial a meniscectomy in patients with degenerative meniscus tear: a secondary analysis of randomized trial. Ann Intern Med. 2016;164(7):449–55.
5. Gamble C, Krishan A, Stocken D, Lewis S, Juszczak E, Dore C, Williamson PR, Altman DG, Montgomery A, Lim P, et al. Guidelines for the content of statistical analysis plans in clinical trials. JAMA. 2017;318(23):2337–43.
6. Schulz KF, Altman DG, Moher D. CONSORT 2010 statement: updated guidelines for reporting parallel group randomised trials. BMJ. 2010; 340:c332.
7. Thorlund JB, Hare KB, Lohmander LS. Large increase in arthroscopic meniscus surgery in the middle-aged and older population in Denmark from 2000 to 2011. Acta Orthop. 2014;85(3):287–92.
8. Hall MJ, Schwartzman A, Zhang J, Liu X. Ambulatory surgery data from hospitals and ambulatory surgery centers: United States. Natl Health Stat Rep. 2010;2017(102):1–15.
9. Thorlund JB, Juhl CB, Roos EM, Lohmander LS. Arthroscopic surgery for degenerative knee: systematic review and meta-analysis of benefits and harms. BMJ. 2015;350:h2747.
10. Khan M, Evaniew N, Bedi A, Ayeni OR, Bhandari M. Arthroscopic surgery for degenerative tears of the meniscus: a systematic review and meta-analysis. CMAJ. 2014;186(14):1057–64.
11. McDermott ID, Amis AA. The consequences of meniscectomy. J Bone Joint Surg Br. 2006;88(12):1549–56.
12. Pajalic KF, Turkiewicz A, Englund M. Update on the risks of complications after knee arthroscopy. BMC Musculoskelet Disord. 2018;19:179.
13. Abram SGF, Hopewell S, Monk AP, Bayliss LE, Beard DJ, Price AJ. Arthroscopic partial meniscectomy for meniscal tears of the knee: a systematic review and meta-analysis. Br J Sports Med 2019. Published online first 22 Feb. https://doi.org/10.1136/bjsports-2018-100223.
14. Winter AR, Collins JE, Katz JN. The likelihood of total knee arthroplasty following arthroscopic surgery for osteoarthritis: a systematic review. BMC Musculoskelet Disord. 2017;18(1):408.
15. Roemer FW, Kwoh CK, Hannon MJ, Hunter DJ, Eckstein F, Grago J, Boudreau RM, Englund M, Guermazi A. Partial meniscectomy is associated with increased risk of incident radiographic osteoarthritis and worsening cartilage damage in the following year. Eur Radiol. 2017;27(1):404–13.
16. Katz JN, Martin SD. Meniscus–friend or foe: epidemiologic observations and surgical implications. Arthritis Rheum. 2009;60(3):633–5.
17. Kirkley A, Griffin S, Whelan D. The development and validation of a quality of life-measurement tool for patients with meniscal pathology: the Western Ontario Meniscal Evaluation Tool (WOMET). Clin J Sport Med. 2007;17(5):349–56.
18. Sihvonen R, Jarvela T, Aho H, Jarvinen T. Validation of the Western Ontario Meniscal Evaluation Tool (WOMET), a meniscal pathology-specific quality-of-life index, for patients with a degenerative meniscus tear. J Bone Joint Surg Am. 2012;94(10):e65–1 -68.
19. Lysholm J, Gillquist J. Evaluation of knee ligament surgery results with special emphasis on use of a scoring scale. Am J Sports Med. 1982; 10(3):150–4.
20. Marx RG. Knee rating scales. Arthroscopy. 2003;19(10):1103–8.
21. Briggs KK, Kocher MS, Rodkey WG, Steadman JR. Reliability, validity, and responsiveness of the Lysholm knee score and Tegner activity scale for patients with meniscal injury of the knee. J Bone Joint Surg Am. 2006;88(4):698–705.
22. Kellgren JH, Lawrence JS. Radiological assessment of osteo-arthrosis. Ann Rheum Dis. 1957;16(4):494–502.
23. Altman RD, Gold GE. Atlas of individual radiographic features in osteoarthritis, revised. Osteoarthritis Cartilage. 2007;15 Suppl A:A1–56.
24. Altman R, Asch E, Bloch D, Bole G, Borenstein D, Brandt K, Christy W, Cooke TD, Greenwald R, Hochberg M, et al. Development of criteria for the classification and reporting of osteoarthritis. Classification of osteoarthritis of the knee. Diagnostic and Therapeutic Criteria Committee of the American Rheumatism Association. Arthritis Rheum. 1986;29(8):1039–49.
25. Hunter DJ, Guermazi A, Lo GH, Grainger AJ, Conaghan PG, Boudreau RM, Roemer FW. Evolution of semi-quantitative whole joint assessment of knee OA: MOAKS (MRI Osteoarthritis Knee Score). Osteoarthr Cartil. 2011;19(8): 990–1002.
26. Ranstam J. Multiple P-values and Bonferroni correction. Osteoarthr Cartil. 2016;24(5):763–4.
27. Emea. ICH Topic E 9 Statistical principles for clinical trials 1998. https://www.ema.europa.eu/en/documents/scientific-guideline/ich-e-9-statistical-principles-clinical-trials-step-5_en.pdf. Accessed 7 Oct 2019.
28. Cummings P. Methods for estimating adjusted risk ratios. Stata J. 2009;9(2): 175–96.
29. Probst P, Zaschke S, Heger P, Harnoss JC, Huttner FJ, Mihaljevic AL, Knebel P, Diener MK. Evidence-based recommendations for blinding in surgical trials. Langenbeck's Arch Surg. 2019;404(3):273–84.
30. Jarvinen TL, Sihvonen R, Bhandari M, Sprague S, Malmivaara A, Paavola M, Schunemann HJ, Guyatt GH. Blinded interpretation of study results can feasibly and effectively diminish interpretation bias. J Clin Epidemiol. 2014;67(7):769–72.

Publisher's Note
Springer Nature remains neutral with regard to jurisdictional claims in published maps and institutional affiliations.
